# Over-Two-Octave Supercontinuum Generation of Light-Carrying Orbital Angular Momentum in Germania-Doped Ring-Core Fiber

**DOI:** 10.3390/s22176699

**Published:** 2022-09-05

**Authors:** Jian Yang, Yingning Wang, Yuxi Fang, Wenpu Geng, Wenqian Zhao, Changjing Bao, Yongxiong Ren, Zhi Wang, Yange Liu, Zhongqi Pan, Yang Yue

**Affiliations:** 1 Institute of Modern Optics, Nankai University, Tianjin 300350, China; 2 Department of Electrical Engineering, University of Southern California, Los Angeles, CA 90089, USA; 3 Department of Electrical & Computer Engineering, University of Louisiana at Lafayette, Lafayette, LA 70504, USA; 4 School of Information and Communications Engineering, Xi’an Jiaotong University, Xi’an 710049, China

**Keywords:** orbital angular momentum, nonlinear optics, supercontinuum

## Abstract

In this paper, we design a silica-cladded Germania-doped ring-core fiber (RCF) that supports orbital angular momentum (OAM) modes. By optimizing the fiber structure parameters, the RCF possesses a near-zero flat dispersion with a total variation of <±30 ps/nm/km over 1770 nm bandwidth from 1040 to 2810 nm for the OAM_1,1_ mode. A beyond-two-octave supercontinuum spectrum of the OAM_1,1_ mode is generated numerically by launching a 40 fs 120 kW pulse train centered at 1400 nm into a 12 cm long designed 50 mol% Ge-doped fiber, which covers 2130 nm bandwidth from 630 nm to 2760 nm at −40 dB of power level. This design can serve as an efficient way to extend the spectral coverage of beams carrying OAM modes for various applications.

## 1. Introduction

Orbital angular momentum (OAM) has gained widespread attention due to its twisted helical phase front and doughnut-shaped intensity distribution. OAM beams have theoretically infinite topological states and unique phase singularity. Therefore, OAM has been applied to a variety of cutting-edge technologies, such as optical communication systems [1,2,3,4,5,6], super-resolution microscopy [7,8], optical sensing [9,10,11], laser material processing [12,13] and imaging [14,15,16,17]. Especially in the field of communication, OAM beams with different topological numbers can form an orthogonally modal set, which can be effectively multiplexed and demultiplexed. Therefore, we can transmit multiple independent beams simultaneously in the same space and frequency band. The spectral efficiency and data capacity of the communication systems can thus be improved by using OAM mode division multiplexing (MDM), since coaxial beams with different OAM states can be efficiently separated. Meanwhile, many methods for the generation of OAM beams have been proposed and experimentally demonstrated by using different types of converters [18,19]. It is of great significance to effectively maintain the OAM beams propagating in the optical fiber. Unfortunately, the OAM beams cannot propagate stably in a conventional fiber, in which the quasi-degenerate modes can be easily coupled to each other. Ring-core fiber (RCF) could potentially solve the problem, as the OAM beams have a similarly ring-shaped intensity profile [20]. OAM beams can be generated from Gaussian beams, but a single OAM beam still has a narrow wavelength range, which limits its application in many fields. Therefore, supercontinuum (SC) generation of OAM beams spanning thousands of nanometers wavelength range is of great significance for applications that require broadband OAM beams.

SC has important applications in many fields, such as optical communication [21], optical frequency comb [22,23] and optical coherence tomography [24,25], which has made it an active research field for decades. One of the most important applications of SC in optical communications is to serve as a multi-wavelength source for ultra-broadband WDM systems [26]. In addition, SC is widely used for all-optical analog-to-digital conversion [27] and TDM-to-WDM-to-TDM conversion [28]. Launching ultrashort pulse with an optical vortex state has been demonstrated to generate SC maintaining optical vortex properties [29]. Moreover, one pioneer research has indicated that an octave-spanning SC of OAM beams could be generated through specially designed optical fibers [30]. In particular, the spectral range of the SC is the key factor to be considered. Recently, SC carrying OAM in fiber has been reported to effectively expand the spectral coverage of the OAM beams [31,32,33,34]. However, most of the core materials used were As_2_S_3_, which is a toxic and harmful substance. In addition, it is not trivial to process and manufacture optical fibers composed of this material [35]. Thus, we choose to use Germania as the core material, which has been widely used in optical fiber manufacturing.

For decades, Germania-doped silica has been one of the most common materials for optical fiber communication due to its excellent physical properties and compatibility with silica glass. Germania-doped silica features lots of excellent properties, such as high mechanical strength, long-term structural stability, low sensitivity to ionizing radiation, low chemical activity, close thermal expansion, etc. These characteristics make it possible to fabricate optical fibers with good geometrical quality and long operation life. In addition, the fabrication of Germania-doped silica fibers has also been demonstrated to be feasible in previous experimental studies [36,37]. Furthermore, this material possesses both low intrinsic absorption and Rayleigh scattering in the near-infrared (IR) spectral range, which leads to very low loss over a wide spectral range.

In this work, we design a 50 mol% Ge-doped ring-core silica fiber supporting OAM modes. We tune its structural parameters to 1 µm inner SiO_2_ radius and 1.8 µm Ge-doped ring width, making the ring-core fiber possessing flat and low dispersion with a small variation of <±30 ps/nm/km from 1040 to 2810 nm. A 70 fs 120 kW secant hyperbolic pulse train with the central wavelength of 1400 nm is chosen to be the input source, which is finally broadened into an over-two-octave SC spectrum carrying the OAM_1,1_ mode with the wavelength from 630 nm to 2760 nm at −40 dB of power level by propagating through a 12 cm long designed 50 mol% Ge-doped ring-core silica fiber. COMSOL Multiphysics software is used to simulate the OAM mode properties, and MATLAB-based generalized pulse-propagation equation is used to simulate the process of SC generation.

## 2. Concept and Fiber Structure

**Figure 1:**
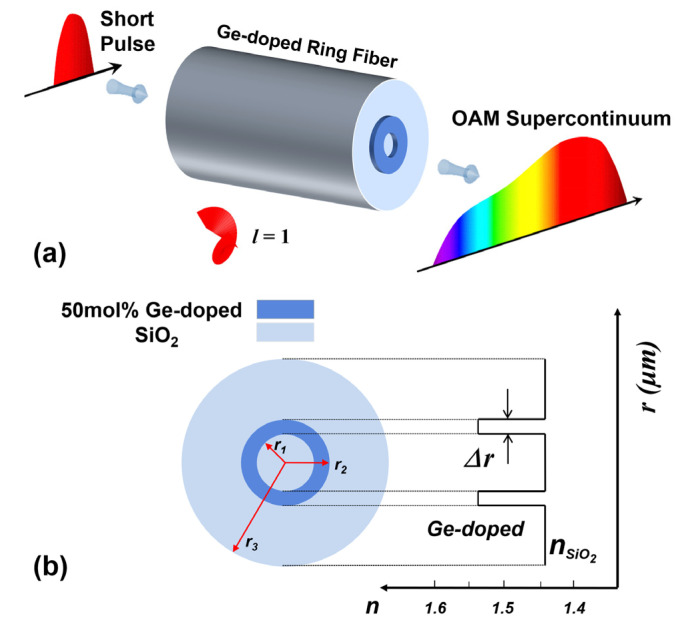
(**a**) Schematic diagram of SC generation; (**b**) Cross-section of the Ge-doped silica fiber.

The schematic diagram of SC generation for the OAM mode is illustrated in Figure 1a. When an intense ultrashort pulse is launched into the optical fiber, the spectrum is widened because of the interaction between the dispersion of the transmission medium and various nonlinear effects. The cross-section of the fiber with a low-index inner SiO_2_ substrate, a high-index Ge-doped ring and a SiO_2_ cladding is shown in Figure 1b.

The material index difference between the silica cladding and Germania-doped annular region is large enough, leading to a large effective refractive index separation between the adjacent modes, which guarantees the modal separation and reduces the corresponding modal crosstalk. Furthermore, this design substantially reduces the modal coupling. Therefore, the choice of fiber materials and the design of fiber structure can perfectly support the propagation of OAM modes. Moreover, the Germania-doped materials have higher nonlinear coefficients than silica over a wide transparent window close to the near-infrared, potentially enabling efficient SC generation in a broad spectral range for the OAM mode. Additionally, we set the cladding diameter to 125 µm, which is identical to the standard single-mode fiber (SMF). Here, our investigation focuses on the SC generation of HE_2,1_, corresponding to the *l* = 1 OAM mode, which is composed of $HE_{2,1}^{even}+i\times HE_{2,1}^{odd}$.

With respect to fabrication, the selection of material and the design of the RCF structure are realistic, and RCFs composed of SiO_2_ and Ge-doped SiO_2_ have been manufactured in practice [38], which has shown good performance on the transmission of OAM modes, especially for the OAM_1,1_ mode.

## 3. Fiber Properties and Dispersion Optimization

In optical waveguides, SC generation is closely related to dispersion conditions. A flat and low dispersion is preferable to achieve SC generation over a wide bandwidth. We calculate the dispersion *D* by Equation (1), which is in the unit of ps/nm/km [39].

(1) $$D=-\frac{\lambda }{c}\frac{d^{2}n_{eff}}{d\lambda^{2}},$$

where *c* is the velocity of light in free space, and *n_eff_* is the effective refractive index of the OAM mode propagating in the designed RCF. To optimize the designed Ge-doped RCF with flat and low dispersion, we investigate the structure parameters, including the doping concentration of Germania, ring width (Δ*r*) and SiO_2_ ring radius (*r*_1_).

**Figure 2:**
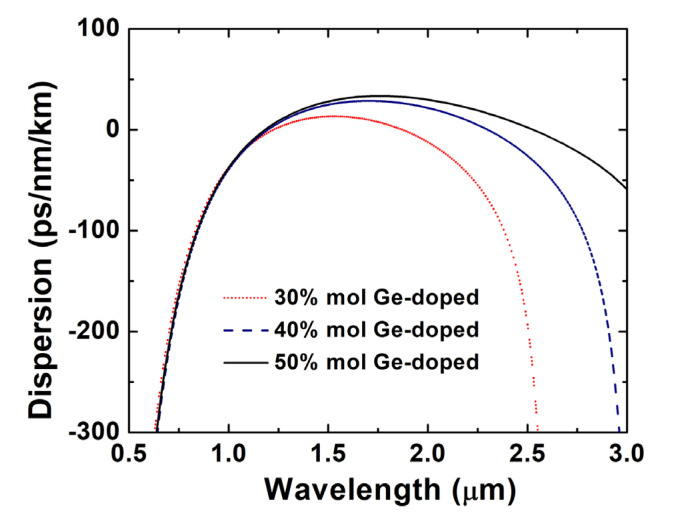
Dispersion-wavelength curves at different Ge-doping concentrations.

The doping concentration of Ge is first optimized, and the dispersion curve of OAM_1,1_ mode is shown in Figure 2. One can see that the dispersion curve moves up with the increase in doping concentration under conditions of the same fiber geometric structure, while the smaller doping concentration leads to a faster-changing dispersion. All the dispersion curves for fibers with different doping concentrations have similar trends, which increase first and then decrease over the wavelength.

It is worth noting that higher doping concentration enables the RCF to support the OAM_1,1_ mode over a wider wavelength range. As higher doping concentration makes the refractive index difference between the ring core and the cladding higher, the OAM mode will have a larger cut-off wavelength with a higher doping concentration, which will directly affect the upper wavelength limit of the SC generation. Finally, the Ge-doped concentration is set to 50 mol%, which can potentially reach the target of generating SC with more than two-octave spectral broadening.

**Figure 3:**
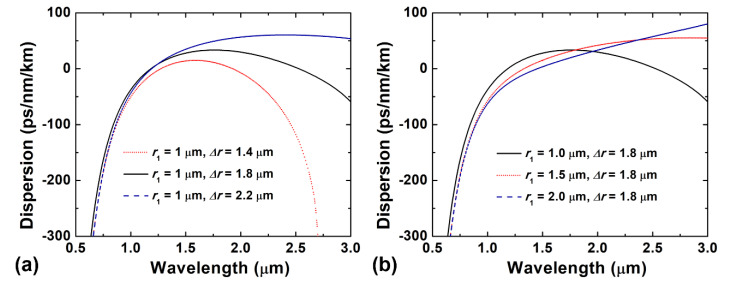
Dispersion-wavelength curves for different (**a**) ring width (Δ*r*) and (**b**) fiber inner SiO_2_ radius (*r*_1_).

Figure 3a illustrates the effect of different ring widths (Δ*r*) on the HE_2,1_ mode dispersion curve. Under the conditions of the same inner silica radius, we can clearly see that the dispersion curve rises as the ring width increases, and the smaller ring width leads to a faster-changing dispersion. Furthermore, we optimize the inner SiO_2_ radius (*r*_1_), which has less effect on the dispersion, as shown in Figure 3b. The upward trend of the dispersion curve becomes more obvious as *r*_1_ increases.

The fiber is relatively dispersive in the short wavelength range due to large material index change. The solid black line in the figure represents the dispersion from 500 nm to 3000 nm of the HE_2,1_ mode in the designed RCF with optimized structure parameters (*r*_1_ =1 μm, Δ*r* =1.8 μm). It can be clearly seen that the optimized RCF structure has a flat and near-zero dispersion profile over a wide wavelength range in the near-infrared region with a total dispersion variation of <±30 ps/nm/km over a 1770 nm bandwidth from 1040 nm to 2810 nm.

**Figure 4:**
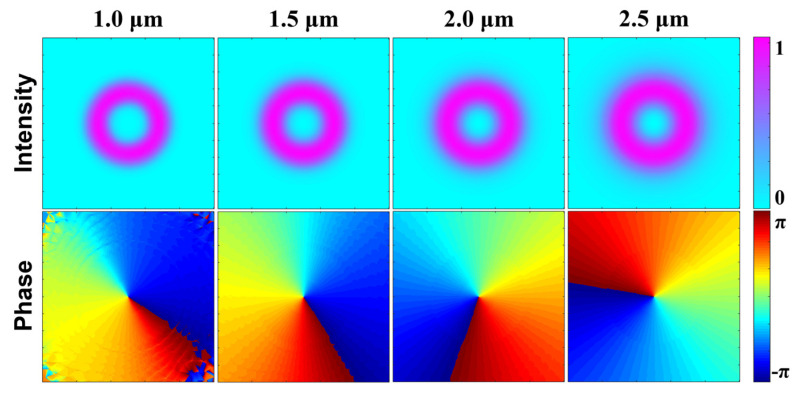
Normalized intensity and phase distributions at different wavelengths of OAM_1,1_ mode supported in the designed RCF.

Figure 4 depicts the intensity and phase distributions of the OAM_1,1_ mode under different wavelengths supported in the designed RCF. The upper and the lower color bars represent the normalized mode field intensity and the phase change of the OAM_1,1_ mode, respectively. These results are calculated through full-vector finite-element method (FEM). One can notice in Figure 4 that the intensity distributions of the OAM_1,1_ mode maintain annular shape constantly, which is well confined within the Ge-doped ring of the fiber, and the effective mode field area increases with wavelength. Meanwhile, the OAM_*l*,1_ mode shows a 2*l*π phase change azimuthally. The azimuthal phase variation of OAM_1,1_ is 2π, corresponding to a topological charge number of 1.

**Figure 5:**
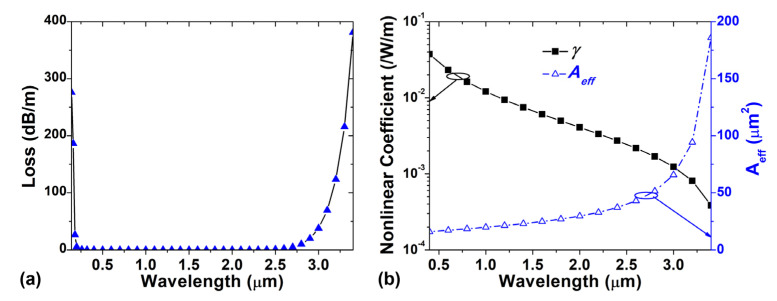
(**a**) Loss, (**b**) nonlinear coefficient and effective mode field area (*A_eff_*) of the RCF with the optimized design.

In the SC generation process, loss is one of the most significant factors. The fiber loss shown in Figure 5a is determined by calculating the imaginary part of the effective refractive index for the OAM_1,1_ mode [40] according to the material loss of silica and Ge-doped silica [41,42]. As the OAM_1,1_ mode cannot be supported normally in the designed RCF for a wavelength larger than 3400 nm, the fiber loss is set to infinity for wavelengths beyond 3400 nm. Nonlinearity is another important parameter that affects the efficiency of the nonlinear process. The nonlinear coefficient (γ) and effective mode area (*A_eff_*) of the OAM_1,1_ mode are displayed in Figure 5b. The nonlinear coefficient decreases as the effective mode area increases with the wavelength, as illustrated in Figure 4.

## 4. Supercontinuum Generation

SC generation is simulated numerically using the generalized pulse-propagation equation, which takes into account the contributions of both the linear effects (dispersion and loss) and the nonlinear effects (Kerr nonlinearity effect, self-steepening effect, etc.) [39].

(2) $$\frac{\partial A}{\partial z}+\frac{1}{2}\left(\alpha \left(\omega_{0}\right)+i\alpha_{1}\frac{\partial }{\partial t}\right)A-i\sum_{n=1}^{\infty }\frac{i^{n}\beta_{n}}{n!}\frac{\partial^{n}A}{\partial t^{n}}=i\gamma \left(1+\frac{i}{\omega_{0}}\frac{\partial }{\partial t}\right)\left(A\left(z,t\right)\int_{0}^{\infty }R(\tau ){\left|A(z,t-\tau )\right|}^{2}\partial \tau \right)$$

where *A* is the electric field envelope, α is the loss coefficient, $\omega_{0}$ is the input pulse frequency, $\beta_{n}$ is the group velocity dispersion (GVD), and *n* is up to 10 in our simulation, τ is the present time frame, R(t) is the nonlinear response function. The functional form of R(t) can be expressed as [39]

(3) $$R(t)=(1-f_{R})\delta (t)+f_{R}\left(\tau_{1}^{-2}+\tau_{2}^{-2}\right)\tau_{1}\exp(-t/\tau_{2})\sin(t/\tau_{1})$$

where $f_{R}$ represents the fractional contribution of the delayed Raman response. The Raman response function coefficients used in Equation (2) are $f_{R}$ = 0.18, *τ*_1_ = 12.2 fs, *τ*_2_ = 83 fs, respectively [43]. The nonlinear refractive index $n_{2}$ for 50 mol% Ge-doped silica is 3.81 × 10^−20^ m^2^/W [44], and a full-vector model is used to obtain the Kerr nonlinear coefficient in the simulation [45,46].

**Figure 6:**
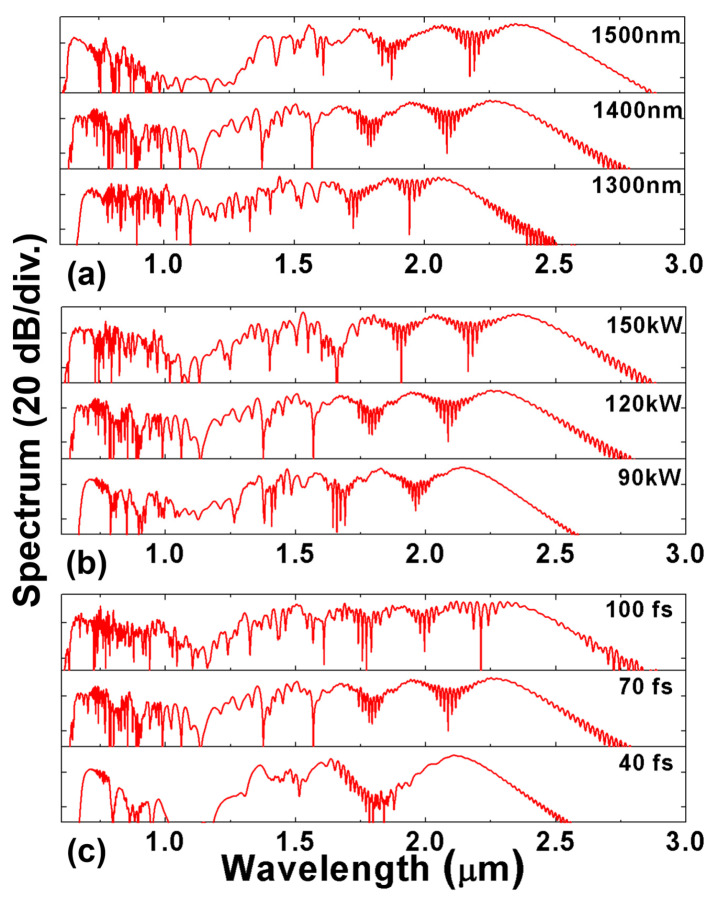
Influence of the initial pulse parameters on SC generation of OAM_1,1_ after a 12 cm designed Ge-doped RCF for different (**a**) *λ*_0_ (*P*_0_ = 120 kW, *T_FWHM_* = 70 fs); (**b**) *P*_0_ (*λ*_0_ = 1400 nm, *T_FWHM_* = 70 fs); (**c**) *T_FWHM_* (*λ*_0_ = 1400 nm, *P*_0_ = 120 kW).

The generated supercontinua in the designed fiber with 1 μm inner SiO_2_ radius and 1.8 μm ring width are shown in Figure 6. We further analyze several key influence factors, including the pump center wavelength, pump peak power and pulse width. The material refractive indices of silica and 50 mol% Ge-doped silica are obtained using the Sellmeier equations in our model [47,48].

The influence of the input center wavelength ($\lambda_{0}$) is illustrated in Figure 6a. The designed 12 cm RCF is pumped by 70 fs 120 kW secant hyperbolic pulses at 1300 nm, 1400 nm and 1500 nm, respectively. One can note that the spectrum obtained by a pump centered at 1400 nm is wider than that at 1300 nm. It reaches more than two-octave spectral broadening, and it is flatter than that at 1500 nm. Furthermore, the output spectrum broadens more widely in the long wavelength range when the pump central wavelength gradually increases. Its spectral power gradually decreases below 1150 nm, which is due to the large normal dispersion for the wavelength shorter than the zero-dispersion wavelength around 1150 nm. This region has a greater requirement for the pump power, and as the pump center wavelength gradually moves away, the power spectral density generated in this region also decreases. Further research found that when the peak power of the input pulse continues to increase, the problem of insufficient pumping at 1150 nm could be solved. In simulations, we found that the spectrum can be easily extended to the short wavelength range as the nonlinear coefficient increases while wavelength decreases. Therefore, pumping at a larger wavelength can further extend the SC into the larger wavelength region. However, the overall quality of the generated SC becomes worse as the pump wavelength moves away from the local minimum of chromatic dispersion.

Then, a 1400 nm 70 fs secant hyperbolic pulse with different input peak power ($P_{0}$) is launched into a 12 cm RCF. The simulation results are shown in Figure 6b. The fiber is pumped by input pulses with different peak powers of 90 kW, 120 kW and 150 kW, respectively. It can clearly be seen that higher peak power will lead to larger SC broadening, while when it reaches a certain range, higher input peak power will induce spectral fluctuation and degrade the SC flatness. An input pulse with a peak power of 120 kW is chosen to achieve a balance between spectral broadness and its flatness. Moreover, we find that symmetrical broadening occurs at lower pump peak power resulting from SPM; nevertheless, the spectrum tends to broaden more toward the longer wavelength region at higher pump peak power as four-wave mixing (FWM) rises in the long wavelength region.

Finally, the impact of the full-width at half maximum ($T_{FWHM}$) of the input pulse is illustrated in Figure 6c. Short pulses of 120 kW with the central wavelength of 1400 nm possessing different pulse widths of 40 fs, 70 fs, 100 fs, respectively, are chosen as the input source launched into the designed RCF. When the peak power is a determined value, the nonlinear effect of the input pulse with a larger pump pulse width is more evident, as it contains more energy and a narrower frequency spectrum, which is the primary reason for the spectral roughness. Thus, we can obviously see that there is more fluctuation in the output SC for an input pulse with a larger pulse width. Furthermore, the output spectrum of the 40 fs input pump pulse is narrower than the others, and the power at 1150 nm is already below −40 dB. This is because the input pulse with shorter duration has a lower total input power and a wider frequency spectrum, which is equivalent to a smaller power spectral density, and thus, it does not provide enough power in the long wavelength region and at around 1150 nm. According to the additional simulation results, the pump power can be boosted to further extend the output SC span for the 40 fs input pulse case.

**Figure 7:**
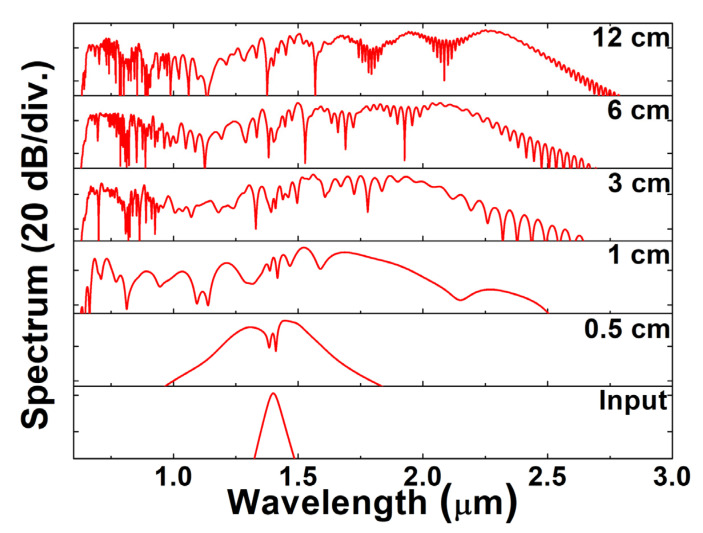
Broadening of the output spectra obtained at different lengths of the designed Ge-doped silica RCF.

We finally chose 120 kW 70 fs pulse at 1400 nm as the pump source, as the corresponding femtosecond laser pump sources are already available [49]. Figure 7 illustrates the process of SC broadening using the 120 kW 70 fs input pulse with central wavelength of 1400 nm after the propagation of 0, 0.5, 1, 3, 6 and 12 cm fiber lengths, respectively. On account of the strong nonlinearity and low dispersion of the designed RCF, the generation of the broadband SC can be achieved in only a few centimeters propagation length. The nonlinear length (*L_NL_*) and the dispersion length (*L_D_*) provide the length scales over which nonlinear or dispersive effects become important for pulse evolution. These two factors can be expressed as [39]

(4) $$L_{NL}=\frac{1}{\gamma P_{0}},L_{D}=\frac{T_{0}{}^{2}}{\left|\beta_{2}\right|}$$

where γ is the nonlinear coefficient, *P*_0_ and *T*_0_ are the peak power and initial width of input pulse, respectively, $\beta_{2}$ is the second-order GVD. The *L_NL_* and *L_D_* for the designed RCF are 0.78 mm and 53 mm, respectively, which means nonlinearity and dispersion act together as the pulse propagates along the 12 cm long RCF. Obviously, the nonlinear effect is stronger than the dispersion effect and plays a leading role in the process of spectrum broadening.

**Figure 8:**
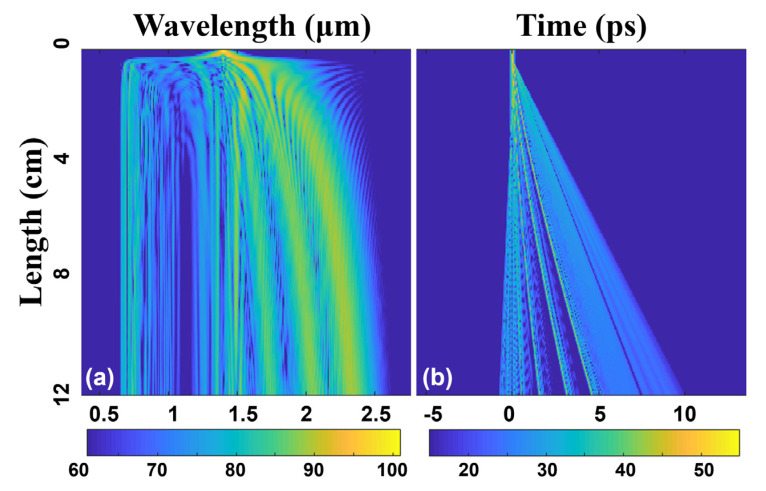
(**a**) **Spectral** and (**b**) temporal evolutions of the pump pulses with OAM_1,1_ mode propagating along the 12 cm optimized RCF.

Figure 8a displays the temporal evolutions of the corresponding SC under different propagation distances, while Figure 8b illustrates the spectral evolutions. After propagating through a 12 cm designed RCF, the SC is extended to approximately 2600 nm wavelength range spanning over two octaves. In the first place, the output pulse is symmetrically broadened around the input pulse wavelength because of the SPM. In the anomalous dispersion region, the high-order soliton effect widens the SC spectrum in the frequency domain and compresses it in the time domain. Then, it splits into fundamental-order solitons, which evolve into several impulse components in the time domain. The broadened spectrum generates dispersive waves in the normal dispersion region. Then, in the normal dispersion region, optical wave breaking (OWB) leads to the generation of new spectrum components [50], and finally, a SC is obtained. The walk-off effect gradually becomes apparent after propagating 1 cm, as illustrated in Figure 8b, which results from the accumulated dispersion. After 8 cm propagation length, the spectrum broadening tends to be stable and smoother, as shown in Figure 8a.

## 5. Conclusions and Perspective

In summary, we design a 50 mol% Ge-doped ring-core silica fiber supporting OAM modes with optimized parameters of 1 µm inner SiO_2_ radius and 1.8 µm Ge-doped ring width, making the ring-core fiber possessing flat and low dispersion with a small variation of <±30 ps/nm/km from 1040 to 2810 nm. It is used for OAM SC generation with a 70 fs 120 kW secant hyperbolic pump pulse centered at 1400 nm. The simulation results show that an over-two-octave SC carrying the OAM_1,1_ mode in the near-infrared region can be generated after 12 cm long RCF, expanding from 630 nm to 2760 nm. This designed RCF can well support the propagation of OAM modes and generate the corresponding SC, which could be utilized for various optical applications. Moreover, it is also prospective that SC carrying higher-order OAM modes can be potentially achieved with the RCF.

## Data Availability

Data underlying the results presented in this paper are not available to the public but can be obtained from the authors upon reasonable request.
